# From Adoption to Continuance: A Longitudinal Qualitative Exploration of University EFL Teachers’ Motivation for Continued Use of GenAI Based on Self-Determination Theory

**DOI:** 10.3390/bs16081396

**Published:** 2026-08-14

**Authors:** Chunhua Mao, Yonghong Zeng

**Affiliations:** 1 Foreign Studies College, Hunan Normal University, Changsha 410081, China; suniamao@hunnu.edu.cn; 2 School of Business Foreign Languages, Hunan Vocational College of Commerce, Changsha 410205, China

**Keywords:** GenAI, university EFL teachers, motivation, continued use, basic psychological needs, self-determination theory

## Abstract

Generative artificial intelligence (GenAI) has been rapidly integrated into higher education, yet current studies largely rely on quantitative frameworks that capture initial adoption, leaving university EFL teachers’ motivational evolution for continued use of GenAI underexplored. To address this gap, this longitudinal qualitative study explores the motivational evolution and psychological need experience of 15 university EFL teachers’continued use of GenAI grounded in Self-Determination Theory (SDT). Data were collected through two rounds of semi-structured interviews over one year and analyzed in NVivo 11 PLUS. Our findings reveal a dynamic evolution of motivation from external regulation to value identification and professional identity integration. Crucially, need satisfaction and need frustration provide important psychological foundations for teachers’ motivation internalization. Notably, while need satisfaction directly facilitates university EFL teachers’ continued use of GenAI, need frustration can actually act as a potential catalyst that facilitates EFL teachers to form long-term continued use of GenAI through iterative reflection and instructional adjustment. This study extends the application of SDT within the GenAI education context, indicating that university EFL teachers’ continued use of GenAI is not merely a process of technology adoption, but also a process of professional growth and professional identity integration. The results provided preliminary implications for teacher professional development, GenAI design and education policy.

## 1. Introduction

The explosive development of generative artificial intelligence (GenAI) is reshaping the global higher education ecosystem at an unprecedented pace (Xi et al., 2025; Qi et al., 2025; Dong & Wang, 2025). Particularly in the EFL teaching field, GenAI tools such as ChatGPT (vGPT-5), Gemini (v2.5) and Deepseek (v3.1) powered by strong capabilities in text creation, language comprehension, diverse multimodal content production and precise interactive response, has shown tremendous potential for EFL teaching empowerment in resource integration, feedback evaluation, personalized learning and immersive dialogue scenario creation (Derakhshan & Ghiasvand, 2024; Wu & Pan, 2025; Wu et al., 2026).

In this context, university EFL teachers have gradually become important practitioners of GenAI education applications and have undergone a transformation from initial “GenAI acceptance” to active “GenAI adoption”. Previous studies have shown that EFL teachers have begun to apply GenAI to various aspects, such as lesson preparation, translation teaching, writing feedback, classroom activity design, and research assistance, and have improved teaching efficiency and resource acquisition capabilities (Derakhshan & Ghiasvand, 2024; X. Liu & Xiao, 2025; Wu & Pan, 2025). However, with the widespread penetration of GenAI in daily teaching, EFL teachers are gradually facing multiple real-life challenges, such as technology illusions, content inaccuracy, academic integrity, data privacy and students’ over-reliance on GenAI (X. Liu, 2025; Moorhouse et al., 2024). These concerns have led to severe technostress and professional identity crises among EFL teachers (X. Zheng et al., 2024; Zeinodin, 2026). Therefore, promoting the smooth transition of EFL teachers from adoption to deep integration of continued use of GenAI has become a core issue that urgently needs to be addressed in the current technological transformation of EFL education.

However, current studies mainly focus on technology adoption, willingness to use, teaching application effectiveness, and teacher attitudes (Baig & Yadegaridehkordi, 2025; Kamsin, 2025). For example, some studies centered on the technical features of GenAI to analyze the perceived ease of use and perceived usefulness of teachers’ willingness to use it (Dehghani & Mashhadi, 2023; Y. Du & Gao, 2022; X. Liu & Xiao, 2025). Similarly, it was reaffirmed by exploring the impact of perceived usefulness, ease of use, or behavioral intention on technology adoption from the Technology Acceptance Model (TAM) and the Unified Theory of Technology Acceptance and Use (UTAUT) (Şimşek et al., 2025; Xue et al., 2024; Zhou & Johar, 2024). What is more, these studies focused more on whether teachers use GenAI and less on why they continue to use it and how this motivation evolves with practical experience (K. Chen et al., 2024; Xue et al., 2024). These studies tended to view GenAI use as a static instrumental rational choice, largely ignoring the deep psychological needs and intrinsic motivation evolution of EFL teachers in the process of technology interaction.

Additionally, most of the existing studies have employed cross-sectional survey designs to examine how factors such as perceived usefulness and perceived ease of use influence teachers’ adoption behaviors (X. Huang & Zhang, 2025; Hazzan-Bishara et al., 2025; B. Li et al., 2026), and largely emphasized teachers’ initial perceptions and short-term adoption of GenAI, while paying insufficient attention to how teachers’ motivation develop and transform during continued use in teaching contexts (Ma & Zhang, 2026; Song et al., 2025; Zhai, 2025). More importantly, few studies have drawn on SDT to examine how basic psychological needs, including autonomy, competence, and relatedness, can jointly contribute to the motivation evolution underlying teachers’ continued engagement with GenAI.

To address this gap, this study adopts a longitudinal qualitative approach to explore the motivation evolution and psychological need experience of university EFL teachers’ continued use of GenAI through the lens of Self-Determination Theory. By tracing teachers’ experiences over time, this study extends the application of SDT to the emerging context of GenAI-supported education and provides preliminary theoretical and practical implications for relevant stakeholders. For EFL teachers, the findings highlight the importance of integrating GenAI into classroom teaching to foster teaching innovation and meaningful human–GenAI collaboration. For policymakers, the study suggests that creating supportive environments that address teachers’ psychological needs may facilitate the continued integration of GenAI into educational practice, although further large-scale and diverse empirical investigations are needed to develop and validate evidence-based policy recommendations. For GenAI developers, this study may provide insights into teachers’ experiences, challenges and expectations, which may inform future product improvement and user-centered technological development.

## 2. Literature Review

### 2.1. Application of GenAI in EFL Education

Recent years have witnessed the rapid development of GenAI, which has significantly reshaped the landscape of EFL education (Derakhshan & Taghizadeh, 2025; F. Huang et al., 2025; Wang et al., 2025). By providing personalized learning, automatic content generation and an interactive learning environment, GenAI has introduced new possibilities for enhancing EFL teachers and students (Wang et al., 2025). Existing studies have primarily highlighted the contribution of GenAI-mediated EFL education to students’ psycho-emotional factors, such as motivation, enjoyment, self-efficacy and social–cognitive abilities (Derakhshan, 2025; Derakhshan & Ghiasvand, 2024; Wang et al., 2025). For instance, Wu (2025) investigated how learners’ creativity and GenAI competence influence their enjoyment and boredom in GenAI-mediated speaking practices (Wu, 2025). What is more, previous studies suggested that GenAI can facilitate students’ language skills in reading (Al-Husban & Alabri, 2025), pronunciation (Kazu & Kuvvetli, 2023), including speaking (F. Huang et al., 2025; Meng & Zou, 2026), writing (Tai et al., 2025) and communication skills (C. Zhang et al., 2024). Despite these benefits and outcomes, existing studies have also identified several challenges associated with GenAI’s use in EFL learning, including overdependence on GenAI, reduction in students’ critical thinking ability (Derakhshan & Taghizadeh, 2025; Tai et al., 2025; Xi et al., 2025) and ethical concerns such as plagiarism and academic misconduct (Valizadeh et al., 2026). Therefore, while the integration of GenAI into EFL education presents substantial opportunities for improving learning outcomes, its potential risks and limitations require careful consideration and appropriate mitigation strategies.

Another important contribution lies in its potential to transform EFL teaching practices. GenAI can significantly reduce teaching workload to save teachers’ preparation time and optimize the teaching preparation process with highly efficient information retrieval and content generation abilities (X. Huang & Zhang, 2025; Tan, 2025). Likewise, GenAI-assisted instructional design and simulated classroom activities can support teachers in refining lesson plans, anticipating students’ responses, and enhancing teaching self-efficacy (Lu et al., 2024). Moreover, empirical studies have demonstrated that GenAI can facilitate personalized and differentiated instruction, assist with assessment, and provide large-scale feedback (Blundell et al., 2025). Meanwhile, GenAI enables teachers to transform theoretical knowledge into practice through support for instructional design, teaching content development and classroom activity creation. Such affordances may promote the transformation of teachers from passive technology recipients to active evaluators and creators by strengthening the integration of technology in teaching (Moorhouse et al., 2024; S. Huang et al., 2025; Kohnke et al., 2025).

So far, a growing strand of study on GenAI-mediated EFL education has focused on GenAI integration, ethical issues and educational injustices. For example, Kurt (2025) explored GenAI’s integration in corpus-based language pedagogy and revealed teachers’ meaningful development of GenAI-supported corpus-based language pedagogy in teaching theory and practice. Furthermore, previous studies have highlighted that GenAI increases the risk of plagiarism and academic misconduct associated with GenAI, emphasizing the importance of establishing ethical guidelines and responsible practices in educational contexts (Hamamra et al., 2026).

Additionally, GenAI literacy has emerged as another important research area in recent years. Jin and Rui (2025) explored GenAI literacy among university EFL teachers in China and found that GenAI literacy exerts a positive impact on all UTAUT dimensions, including behavioral intention, performance expectance, effort expectancy, and facilitating conditions. Similarly, another study further reaffirmed this by revealing how EFL learners’ self-efficacy and AI literacy jointly influence their engagement (W. Zheng, 2026). What is more, Wang et al. (2025) developed and validated a GenAI literacy scale for the Chinese EFL context, providing an important instrument for assessing users’ competencies in interacting with GenAI (Wang et al., 2025).

Although current studies have substantially advanced understanding of the application of GenAI in EFL education, most empirical studies still pay insufficient attention to the subjectivity of EFL teachers. As key agents who translate GenAI technologies into pedagogical practices, EFL teachers play a central role in determining the effectiveness and sustainability of GenAI integration. Therefore, investigating the dynamic interaction between EFL teachers and GenAI, particularly motivational evolution and psychological need experience underlying teachers’ continued engagement, represents an important direction for future research.

### 2.2. University EFL Teachers’ Use of GenAI

With the explosive iteration of GenAI, GenAI is reshaping preparation, classroom implementation, feedback evaluation and professional development of EFL teaching, making EFL teachers important actors in the intelligent transformation of EFL education (Kim, 2024; Zou et al., 2025).

Early research on EFL teachers’ use of GenAI focused on teachers’ cognition, attitude and willingness to accept GenAI. The Technology Acceptance Model (TAM) has been widely used to explain why EFL teachers are willing to adopt GenAI. For instance, perceived usefulness and perceived ease of use are regarded as important factors influencing EFL teachers’ adoption of GenAI (Wu & Dong, 2025; Lu et al., 2024; Dehghani & Mashhadi, 2023). The more EFL teachers find GenAI useful, convenient, and manageable, the stronger their acceptance intention becomes (Y. Du & Gao, 2022). In a similar way, Dehghani and Mashhadi (2023) explored the factors that influence the acceptance of ChatGPT among Iranian EFL teachers based on the TAM and pinpointed that ChatGPT’s perceived usefulness and perceived ease of use had significantly influenced the acceptance and adoption among Iranian EFL teachers (Dehghani & Mashhadi, 2023). In addition, Hazzan-Bishara et al. (2025) complemented TAM theory by integrating psychological factors, indicating that teachers’ readiness to accept credible GenAI information positively affected perceptions of GenAI usefulness and further strengthened their intention to use it. When EFL teachers believe that GenAI can improve lesson preparation efficiency, reduce workload, or optimize classroom design, their willingness to use it significantly increases. At the same time, EFL teachers’ GenAI anxiety, ethical concerns and doubts about the reliability of GenAI may also inhibit their willingness to use it (Belda-Medina & Kokošková, 2024; X. Liu & Liu, 2025; Y. Zhang, 2025).

Another strand of research has focused on how GenAI empowers EFL teachers in EFL teaching (Wang et al., 2025). Likewise, GenAI-assisted instructional design and simulated classroom activities can optimize teaching plans and simulate student responses, enhancing their teaching confidence and ultimately improving their self-efficacy (X. Huang & Zhang, 2025; Tan, 2025; Lu et al., 2024). What is more, GenAI can help teachers transform theoretical knowledge into practice by assisting in instructional design, teaching content development and classroom activity creation and promote the transformation of teachers from passive technology recipients to active evaluators and creators by enhancing the integrating ability of technology in teaching (Moorhouse et al., 2024; S. Huang et al., 2025; Kohnke et al., 2025). In another study, Zhi and Wang (2024) highlighted the potential implications of AI technologies to foster positive teacher–student relationships in encouraging students’ willingness to communicate. Although most EFL teachers acknowledged the value of GenAI in improving teaching efficiency, promoting personalized teaching, and enriching teaching resources, they also expressed concerns about content accuracy, academic integrity, data privacy and students’ over-reliance on GenAI (X. Liu, 2025; Wang et al., 2025).

So far, a concomitant number of studies have been conducted on EFL teachers’ use behavior of GenAI from more complex theoretical frameworks. For instance, some studies have integrated social–cultural theory, teacher beliefs, and emotional factors based on traditional TAM to explain the differences in teacher participation in GenAI practice in different educational contexts (Cong-Lem et al., 2024; Mostafavi et al., 2025; Wang et al., 2025). What is more, psychological factors such as teachers’ beliefs, emotion regulation and professional identity play a key role in GenAI technology adoption decisions (Dishari, 2026; Guan et al., 2024; Y. Li & Khairani, 2026).

Although existing studies have clarified the factors influencing teachers’ adoption of technology, they still focus on “adoption” or “acceptance” with a cross-sectional survey design and lack explanation and empirical support for the internalization and development of motivation over time, indicating the need for a motivational framework such as SDT.

### 2.3. Self-Determination Theory

Self-Determination Theory (SDT), proposed by Deci and Ryan (1985), is a classic theoretical framework for explaining individuals’ motivation (Deci & Ryan, 2015; Ryan & Deci, 2018). According to SDT, individuals’ engagement in a behavior is determined not only by what they do but by why they engage in that behavior (Deci & Ryan, 2000). It proposes a continuum of motivation along amotivation-controlled motivation-autonomous motivation, suggesting that external regulations can gradually become internalized when individuals perceive personal value and integration with their goals (Ryan & Deci, 2000a; Deci & Ryan, 2000). Amotivation represents a state in which an individual lacks the intention to regulate behavior and the driving force for action. With different level of internalization, the less internalized form of motivation is called controlled motivation covering external regulation (driven by external rewards or punishments) and introjected regulation (forced to take action to avoid guilt or maintain self-value), while the more internalized form of motivation is called autonomous motivation consisting of intrinsic motivation (to act out of interest and the pleasure of the task itself), identified regulation (to recognize learning value and actively identify with goals) and integrated motivation (highly internalized from of internalizing behavior as part of their self-belief system) (Ryan & Deci, 2018, 2020). SDT suggests that autonomous motivation is associated with adaptive outcomes, such as higher goal persistence, stronger learning enjoyment, and higher academic achievement, while controlled motivation is linked to maladaptive outcomes, such as learning anxiety, job burnout and lower academic performance (M. Liu & Oga-Baldwin, 2022; Noels et al., 2000). A meta-analytic study has supported the continuum motivation of SDT, suggesting that different motivational regulations represent qualitatively distinct but interconnected forms of motivation rather than isolated categories (Howard et al., 2017).

SDT further proposes that an individual’s development is closely associated with the satisfaction and frustration of three basic psychological needs: autonomy, competence, and relatedness (Ryan & Deci, 2000a, 2000b; Vansteenkiste & Ryan, 2013).

Individuals’ basic needs comprise the needs for autonomy, competence and relatedness. Autonomy concerns experiencing one’s actions as freely chosen without external pressure or control; competence pertains to feeling effective in interactions with the social environment and capable of expressing and exercising one’s capacities; and relatedness refers to feeling connected to others, cared for and being cared for within a social group (Ryan & Deci, 2020). According to SDT, when an individual’s basic psychological needs are satisfied, they are more easily motivated intrinsically and the internalization process of external regulation is also effectively promoted, exhibiting more continued and high-quality behavioral engagement (Ryan & Deci, 2020; W. Du et al., 2023; Y. Li et al., 2024). Conversely, when basic psychological needs are frustrated, an individual’s intrinsic motivation may be undermined and individuals may become more dependent on controlled motivation, potentially leading to negative outcomes such as anxiety, fatigue and reduced engagement (Ryan & Deci, 2020; Derakhshan & Ghiasvand, 2024).

Recently, relevant research in educational technology has verified the effectiveness of SDT in explaining teachers’ motivation for technology use, providing empirical support for the theoretical selection in this study. As Chiu et al. (2024) pointed out, the supportive environment given by the school can enhance teachers’ willingness to continuously integrate technology in the classroom by satisfying their basic psychological needs (Chiu et al., 2024). Correspondingly, Zhu and Xia (2025) explored the impact of teachers’ psychological needs satisfaction on their intention to integrate technology behavior based on the SDT framework, using GenAI literacy and self-efficacy as mediating variables. Collectively, these findings suggest that teachers’ technology-related behaviors are not solely determined by tool attributes or external conditions, but are deeply shaped by their psychological need experiences and motivational processes.

However, existing research has primarily focused on teachers’ initial technology acceptance or intention to use, with limited attention paid to how teachers’ motivation develops and transforms through continued engagement with emerging technologies over time. In particular, little is known about how university EFL teachers’ motivations for continued use of GenAI evolve through accumulated teaching experiences and how the satisfaction and frustration of psychological needs at different stages contribute to this motivational transformation. Accordingly, SDT provides a valuable theoretical lens for investigating the dynamic evolution of EFL teachers’ motivation during continued use of GenAI. Exploring this process may further extend the explanatory power of SDT within the emerging context of GenAI-supported EFL.

### 2.4. Purpose and Significance of the Study

Based on the above literature review, this study aims to explore how university EFL teachers’ motivation for continued use of GenAI evolves over time and how the motivational evolution is jointly shaped by the satisfaction and frustration of the three basic psychological needs within the framework of SDT. With the two rounds of interviews, the study seeks to uncover the dynamic motivational mechanism underlying continued use of GenAI.

A longitudinal qualitative design was adopted because SDT posits that motivation is a dynamic process involving continuous internalization and integration (Ryan & Deci, 2020). An individual’s motivation may undergo continuous changes as their basic psychological needs are satisfied or frustrated, gradually evolving from external regulation to intrinsic motivation. Therefore, the motivation evolution for university EFL teachers’ continued use of GenAI represents an ongoing developmental process, rather than a fixed stable state captured at a single point in time. Compared with cross-sectional studies, longitudinal qualitative research can better capture the continuous process through which motivation becomes internalized, and teachers’ psychological experiences related to basic needs develop and transform over time.

The study contributes to the existing literature in several ways. Theoretically, it extends SDT to the context of GenAI-supported EFL education, revealing the dynamic evolution and psychological mechanisms of motivation for EFL teachers’ continued use of GenAI. Methodologically, it adopts a longitudinal qualitative design, addressing the gap in existing research that has predominantly relied on cross-sectional approaches and providing a dynamic perspective for understanding university EFL teachers’ continued use of GenAI. Practically, the findings provide preliminary insights for EFL teachers’ professional development, education policymakers and GenAI developers. Specifically, the study seeks to address the following questions:

**RQ1.**  *How has university EFL teachers’ motivation for continued use of GenAI evolved over time?*

**RQ2.**  *How do the satisfaction and frustration of basic psychological needs contribute to the motivational evolution?*

## 3. Methodology

### 3.1. Participants

The study employed purposive sampling to select EFL teachers from diverse backgrounds who belonged to the target population with experience using GenAI, aiming to collect both rich and diverse data (Merriam & Tisdell, 2016). Participants were required to meet three criteria: (a) willingness to participate in two rounds of interviews; (b) having prior experience using GenAI; and (c) varied demographic characteristics and professional backgrounds, to achieve sample diversity andensure the inclusion of EFL teachers with diverse teaching experiences and perspectives. Finally, we recruited 15 EFL teachers from different universities or colleges in China with certain diversity in their backgrounds, such as age, gender, academic disciplines, institution and discipline, as presented in Table 1.

It should be noted that although the sample size of this study (*N* = 15) is sufficient to support the longitudinal qualitative research conducted, the study does not aim to achieve statistical representativeness. Instead, the sampling strategy prioritizes cases with information-rich cases and seeks to achieve maximum variation across key demographic dimensions in order to capture the diverse experiential diversity of EFL teachers in this specific context (Patton, 2015). Therefore, the findings should be interpreted as an in-depth explanation of EFL teachers’ experience of continued use of GenAI in specific educational contexts, rather than a universal broad generalization for all groups of EFL teachers in universities.

### 3.2. Interview Protocol

A longitudinal qualitative design was adopted because the purpose of this study was not merely to identify teachers’ motivations for using GenAI, but to explore the dynamic motivation evolution and psychological need experience of EFL teachers during continued use of GenAI. Unlike cross-sectional approaches that capture motivation at a single point in time, longitudinal qualitative research enables researchers to examine processes of change, continuity, and transformation within participants’ experiences (Saldaña, 2003; Thomson & Holland, 2003).

Two rounds of semi-structured interviews were conducted by the first author via WeChat (online) or in person (offline), tracking the same participants over time in February–March 2025 and February–March 2026, respectively. These interviews were supplemented by the collection of teaching materials generated through GenAI to provide additional contextualized evidence of teachers’ actual practices.

Before formal data collection, the researchers repeatedly refined the interview protocol through multiple rounds of discussions and conducted a pilot study to test the appropriateness and feasibility of the interview protocol. The pilot study invited three university EFL teachers who met the inclusion criteria but were not included in the formal sample. It focused on assessing the clarity, comprehensibility, logical sequence, and practicality of the interview questions and interview process. Participants provided feedback on the wording of questions, relevance of interview content, and effectiveness of interview prompts based on their practical experience of continued use of GenAI.

Based on the pilot results, the researcher further refined the interview protocol by clarifying ambiguous statements, adjusting the sequence of questions, and supplementing follow-up questions on motivation evolution and basic psychological needs experience, in order to obtain richer and deeper data. The final semi-structured interview outline was then applied during formal data collection, which enhanced the consistency of interview implementation and the credibility of the research findings.

The interview questions differed between the two rounds. The first round was designed to capture participants’ initial perceptions and practices, while the second round included three targeted follow-up and supplementary questions developed iteratively based on the first-round responses. These questions aimed to explore the process of evolution and underlying reasons for any changes in motivation and practices as well as the underlying reasons for such changes over the one-year period, thereby enhancing the longitudinal depth of the study. All questions from the two rounds of interviews were provided in Appendix A.

The author obtained ethical approval from the university’s human research ethics committee. With participants’ informed consent, all interviews were audio-recorded and transcribed verbatim for subsequent analysis. Each interview was held in Chinese and lasted about 30 min on average. The participants from different colleges or universities were encouraged to provide rich and detailed accounts of their experiences with continued use of GenAI.

### 3.3. Data Collection

Creswell’s (2013) interview guidelines were followed during the two rounds of interviews. The first round of interviews took place in February–March 2025, focusing on EFL teachers’ subjective experience during the early stages of GenAI use, including initial motivations and practical reasons for continued use of GenAI as well as their initial perception of GenAI. The second round of interviews was conducted in February–March 2026, aiming to track changes in EFL teachers’ experiences of continued use of GenAI.

Additionally, this study also collected supporting materials supported by participants for data triangulation, such as fragments of teaching plans generated by participants using GenAI as well as students’ essays andhomework grading feedback records.

To enhance the trustworthiness of the collected data, each round of interviews followed four steps. Firstly, the researchers designed the semi-structured interview outline based on the core concepts of SDT and the existing relevant literature, and then submitted the outline to three experts in EFL education with extensive experience in qualitative research for review. Experts provided revision suggestions in terms of problem appropriateness, theoretical appropriateness, expression clarity and logical sequence. The researcher made the first round of revisions to the outline based on the feedback, enhancing the accuracy of the interview questions. Secondly, a pilot test was conducted with three EFL teachers from the authors’ institution who met the requirements of the study but did not participate in formal interviews. In line with the specific feedback on question expression, understanding difficulty and interview duration, the researcher adjusted the wording of some questions and optimized the interview questions to better reflect the teachers’ real experience, resulting in the final version of the interview outline. Subsequently, the semi-structured interviews were administered to fifteen EFL teachers from 5 universities and 5 colleges. The sample selection took into account the gender, teaching experience, professional title, and type of institution in order to obtain diverse and rich research perspectives. All participants signed an informed consent form and were clearly informed of the study purpose, data usage, anonymity and confidentiality terms. Moreover, all interview materials were anonymized and coded as a substitute for personal information. The researcher has no prior relationships or conflicting interests with the participants, ensuring the objectivity of the study. Finally, with the time and modality of the interview discussed and agreed upon with all the participants, the interviews were conducted via WeChat software or in person. During the interview process, participants were encouraged to showcase teaching materials and student feedback logs generated with GenAI to enrich and contextualize their accounts of GenAI use. The interviews were audio-recorded and transcribed word-for-word for thematic analysis, as described in the subsequent section.

### 3.4. Data Analysis

The supporting materials were not encoded as independent datasets for theme development, but used to contextualize participants’ narratives, examine the consistency between participants’ accounts and teaching practices, and support triangulation when explaining themes. When there are discrepancies between interview records and the supporting materials, the research team will discuss them until consensus is reached. This process enhanced trustworthiness.

**Figure 1:**
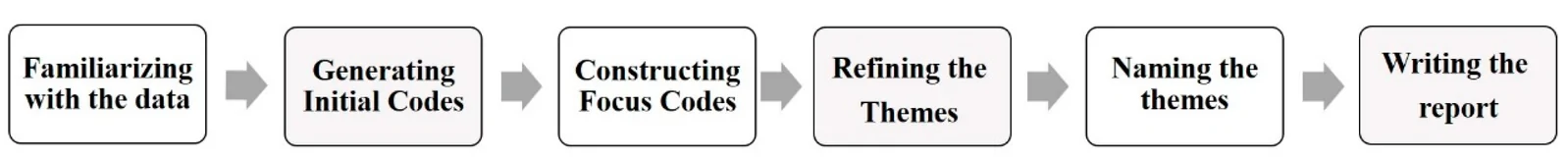
Thematic analysis steps.

**Figure 2:**
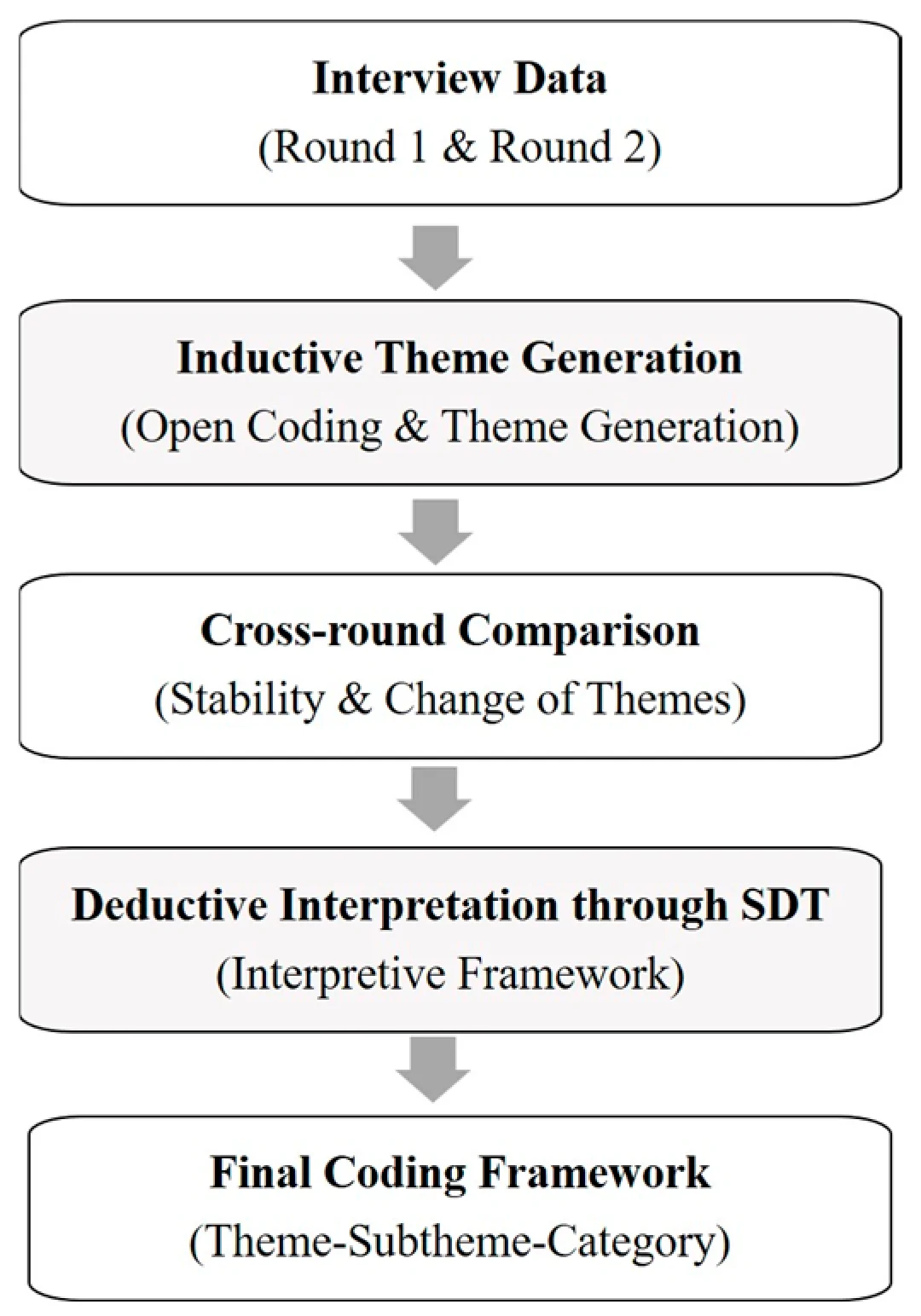
Analytical process of the longitudinal thematic analysis.

The study adopted the thematic analysis method put forward by Braun and Clarke (2006) (Figure 1) with NVivo 11 Plus to analyze two rounds of interview data, in order to reveal the dynamic evolution of motivation and basic psychological needs of university EFL teachers’ continued use of GenAI over a one-year period. Considering the longitudinal design of this study, two rounds of interview data were first independently analyzed, followed by cross-round comparisons to identify the evolving trajectories of motivation and psychological needs of EFL teachers during their continued use of GenAI. The entire analysis process follows the two-staged “inductive-to-deductive” analysis strategy (Goldfarb et al., 2021) and maintained a complete audit trail, recording key decisions such as coding, category integration, theme revision, and theoretical mapping to improve the transparency of the analysis process (Figure 2).

Inductive thematic analysis was adopted in the first stage of analysis. The researchers first repeatedly read the interview transcripts to immerse themselves in the data and used analytical memos to document preliminary obsercations. Subsequently, line-by-line open coding was performed on the transcripts to generate initial codes derived from participants’ original expression. By continuously comparing the similarities and differences in coding within and among participants, similar coding was integrated into preliminary categories, and further summarized to form emerging themes reflecting EFL teachers’ continued use of GenAI. During the process of theme formation, the researchers continuously examined the consistency between emerging themes and the original data, and continuously revised and refined the theme structure through repeated discussions.

Deductive thematic analysis was conducted in the second stage. After completing the inductive analysis, the emerging themes were further interpreted through the theoretical lens of SDT. SDT was not used as a prior encoding framework, but was used to explain its theoretical significance after the themes were formed. Researchers focus on examining how the themes formed by induction correspond to different types of motivations, including external regulation, introjected regulation, identified regulation, integrated regulation, and intrinsic motivation, as well as the satisfaction and frustration of the three basic psychological needs of autonomy, competence, and relatedness. For themes that cannot be fully incorporated into the SDT framework, they are still retained to ensure that the analysis can fully reflect the true experiences of participants, rather than being limited by theoretical frameworks.

After completing independent analysis of two rounds of interviews, the researchers further conducted cross-temporal comparisons to analyze the stability and variability of themes across time. This process enabled the identification of persistent, emerging and changing themes and facilitated the construction of an evolutionary trajectory of university EFL teachers’ motivation and basic psychological needs related to continued use of GenAI.

To enhance the credibility of the research results, two researchers independently coded about 25% of the interview data and discussed coding boundaries, category division, and theme naming until a consensus was reached. Subsequently, the finalized coding framework was applied to all interview materials, ultimately resulting in a three-level coding structure consisting of “theme–subtheme–category”. Figure 2 presents the overall analysis process of the study.

In addition, to further enhance the credibility of the findings, the researchers continuously reviewed the original interview transcripts throughout the entire analysis process, ensuring that the finalized themes accurately reflected the participants’ real experiences and minimizing the potential influence of theoretical frameworks on inductive analysis.

It should be noted that this study adopted a cross-round thematic analysis, with the analysis unit being the shared meaning patterns emerging from participants’ accounts, rather than individual participants’ development trajectories. Given that the aim of this study was to reveal the shared motivational patterns and changes in basic psychological needs of university EFL teachers in their continued use of GenAI, the analysis focused on the themes’ structures, contents, and meanings between different rounds, rather than establishing an independent motivational development history for each teacher. Individual differences among participants were still preserved during the coding process, and commonalities and differences among themes were identified through cross-participant comparisons.

### 3.5. Trustworthiness

According to the qualitative research quality trustworthiness criteria proposed by Lincoln et al. (1985), the study enhanced research rigor through four dimensions: credibility, transferability, dependability and confirmability. To establish credibility, this study conducted longitudinal data collection through two rounds of semi-structured interviews and incorporated the supporting materials, such as fragments from a lesson plan generated by GenAI and student homework correction records for triangulation to corroborate interview accounts and enhance the credibility of the findings. To enhance transferability, the study provided a detailed contextual description of the research context, participant characteristics, data sources and analysis process to help readers assess the applicability of research findings to similar contexts. In addition, the study maintained a relatively complete record of the research process, including interview protocols, transcripts, coding frameworks, analysis memos and thematic development processes, thereby enhancing dependability. Finally, to achieve confirmability, the study incorporated participants’ original quotations and the supporting materials as evidence in the results. Moreover, researchers engaged in continued reflection and examination of theoretical assumptions and explanations to ensure that research findings were grounded in the empirical data. The supporting materials were treated as a component of data triangulation and were used to corroborate interview evidence rather than generate themes.

## 4. Results

### 4.1. Initial Motivation and Basic Psychological Needs Experience in the First Round of Interviews (February–March 2025)

#### 4.1.1. Motivational Types in the First Round of Interviews (February–March 2025)

In the first round, most university EFL teachers’ motivation for continued use of GenAI was still in the initial adoption stage. With their motivation for continued use of GenAI primarily characterized by external regulation and identified regulation, some participants also demonstrated elements of introjected regulation and weak intrinsic motivation. However, integrated regulation has not yet been fully formed (Table 2).

##### External Regulation

External regulation decribes behaviors regulated by external contingencies, such as rewards and punishments, and constitutes the most controlled and non-autonomous form of motivation within SDT (Ryan & Deci, 2020). In the present study, external regulation was mainly reflected in two categories: improving work efficiency and dealing with teaching and writing tasks.

The most frequently mentioned motivation was improving work efficiency and saving time. As T1 revealed, “*GenAI can help us resolve the difficulties in work to improve our working efficiency and save time*”. Some EFL teachers use GenAI to deal with teaching and writing work such as analyzing test papers and writing materials. T9 argued that “*we always use GenAI to writing some regular text materials such as some reports and summary*”.

EFL teachers’ external regulation for continued use of GenAI in the first round was mainly reflected by improving work efficiency and reducing work burden. University EFL teachers primarily positioned GenAI as a tool to support daily work, and their motivation remained primarily driven by external outcomes.

##### Introjected Regulation

Introjected regulation refers to a partially internalized form of extrinsic motivation driven by internal pressures experienced by individuals, such as guilt or shame, still influenced by external standards (Ryan & Deci, 2020). Introjected regulation was reflected in two categories: keeping up with the times and following peer choices.

To keep pace with the times is an important reason for EFL teachers to use GenAI. As T14 mentioned in the interviews, “*In the AI era, GenAI has become indispensable for educators. It is imperative to keep pace with technological advancements*”. Many university EFL teachers mentioned that they followed peer choices. As T4 noted, “*I use Doubao for my students’ recommendation*”. Another teacher supported this by declaring that “*My colleague recommended it to be very useful, so we tried using it (T1)*”.

EFL teachers’ motivations for continued use of GenAI at this stage shared a common orientation toward maintaining professional competitiveness and adapting to the development of GenAI. Their motivation had begun to internalize initially, but still mainly stayed at the stage of “should be used”, not yet forming a deep recognition of the educational value of GenAI.

##### Identified Regulation

Identified regulation refers to a form of autonomous extrinsic motivation in which individuals consciously recognize and personally endorse the value of an activity, thereby experiencing a greater sense of volition and willingness to engage in the behavior (Ryan & Deci, 2020). In the present study, it was reflected in three categories: sparking new ideas and broadening perspectives, and enhancing teaching design and scientific research.

Many EFL teachers recognized the practical value of GenAI in sparking new ideas and broadening perspectives. T8 gave her opinion by stating: “*It can help solve something I have no idea and provide some new information from other perspective*”. Some other EFL teachers viewed GenAI as a supportive resource for enhancing teaching design and scientific research. As stated by T11, “*I will use GenAI to expand teaching ideas, setting up exercises and optimizing teaching plan*”. Another teacher focused on the use of GenAI in scientific research by stating, “*GenAI can be used to expand the idea of scientific research and analyze data more professionally*” (T3).

EFL teachers have begun to go beyond simple efficiency needs and gradually recognize the value of GenAI in promoting instructional design, research support, and expanding thinking. Their motivation for continued use of GenAI has shown a higher degree of autonomy and internalization tendency.

##### Intrinsic Motivation

Intrinsic motivation refers to engaging in activities because they are inherently interesting, enjoyable, and satisfying, rather than because of external pressures or instrumental outcomes (Ryan & Deci, 2000a).

Some EFL teachers mentioned that their initial engagement with GenAI was driven by obvious curiosity and a desire to explore the capabilities of this emerging technology. As declared by T4, “*I like to try new things. GenAI, as a new technology tool, is very attractive for me*.” T9 reiterated the same claim, saying, “*Out of curiosity, this new technology itself is also attractive*”. Many EFL teachers also showed interest in exploring the different usages. T2 stated that “*I want to know how much help AI can provide in teaching and academic research*”.

Intrinsic motivation was still in its infancy. Teachers’ continued use of GenAI was more out of curiosity and exploratory interest in new technology. Although this interest has not yet become the primary driving force for continued use, it exhibits an early shift from instrumental engagement toward enjoyment and interest in the process of technology exploration.

Overall, teachers’ motivation for continued use of GenAI in the first round of interviews showed a trend of gradually developing from external regulation to autonomous internalization. Teachers initially used GenAI to improve efficiency and accomplish practical tasks and professional adaptation pressures encouraged them to recognize the value of GenAI for teaching, research, and personal development. Meanwhile, emerging curiosity-driven exploration facilitated the early development of more autonomous forms of motivation.

#### 4.1.2. Basic Psychological Needs Experience in the First Round of Interviews (February–March 2025)

Most university EFL teachers’ motivation for continued use of GenAI was closely linked to theinitial satisfaction of the three basic psychological needs: autonomy, competence and relatedness. However, frustratin of these needs among teachers constrained the internalization of their motivation (Table 3).

##### Autonomy Experience

Most university EFL teachers have experienced autonomous satisfaction through flexibly preparing teaching materials, efficiently acquiring information and designing classroom assignments through the use of GenAI. As noted by T7: “*GenAI can quickly offer some relatively accurate information and data according to your prompts, making my lesson preparation much easier*”. However, some university EFL teachers also clearly experienced frustration due to insufficient proficiency in advanced GenAI applications and limitations in mobile device access when using GenAI. T1 pointed out that Insufficient mastery of GenAI restricts autonomous action by declaring, “*When it comes to this more complex skill such as drawing, I haven’t used it because I haven’t mastered this technical ability well enough*”.

Most EFL teachers’ autonomy needs in the first round of interviews demonstrated a combination of satisfaction and frustration. Although teachers can use GenAI to enhance their teaching autonomy and resource acquisition efficiency, their experience of autonomy satisfaction remained affected by insufficient technological competence and institutional constraints. Therefore, autonomy need has been initially met, but stable autonomy had not been fully established, and teachers’ motivation for continued use of GenAI remained at an early stage of internalization.

##### Competence Experience

Most university EFL teachers generally perceived that GenAI enhanced their competence satisfaction by improving work efficiency, supporting lesson planning and providing preliminary assistance in academic activities. As noted by T4, “*I use AI to help grade my oral homework. After inputting into AI, it will help me analyze the errors in vocabulary usage and collocation in this expression*”. T3 also mentioned his sense of competence with GenAI in academic research, saying “*GenAI can help me improve my efficiency in reading literature. Some literature takes me an hour to read on my own, but with the help of AI, sometimes I can read five or six articles in an hour*”. Competence satisfaction provided positive experiences that supported teachers’ continued use of GenAI: teachers felt more capable of managing routine teaching and research tasks, and this experience facilitated them to continue using GenAI.

Some teachers also experienced competence frustration when they frequently encountered problems such as difficulties in using GenAI tools, receiving inaccurate outputs and being unable to perform more advanced GenAI-supported tasks. T1 declared that “*I don’t know how to use this technology to generate PPT or Mind map yet”.* And T8 also stated*, “The materials generated by GenAI are relatively fixed and structured, which is a problem*”. T10 confirmed that by saying: “*I don’t think the lesson plan it generates really meets my needs…I still need to put in a lot of effort to process it further*”. Experiencing competence frustration prevented teachers from using GenAI on safer, simpler, and more repetitive tasks, restricting their continued use to a certain degree.

In sum, most EFL teachers’ competence needs reflected a coexistence of satisfaction and frustration. GenAI enhanced teachers’ sense of competence in completing teaching and research tasks, but its output quality and teachers’ limited technological skills constrained its deeper application. Therefore, teachers’ competence needs have been initially met, but stable competence support has not yet been established, and their continued use remains concentrated at the basic application level.

##### Relatedness Experience

Relatedness satisfaction began to emerge when EFL teachers used GenAI to provide personalized feedback, promote classroom discussions and increase student engagement. T3 stated that “*with the help of AI, homework problems will receive more detailed feedback, students’ interest in learning will increase, and they will know where to improve*”. At this stage, GenAI has begun to help teachers and students form a new mode of interaction and has already laid the foundation for deeper teaching empowerment in the future.

Relatedness frustration was evident when EFL teachers perceived that the use of GenAI could contribute to students’ over-dependence on GenAI, exposure to inaccurate outputs and reduced authenticity of learning experiences. As T1 said, “*I feel that students used better than me, and cheating in homework is more severe*”. T9 also expressed the same concern: “*If students blindly rely on it, they will not have independent thinking on their own*”. T2 expressed the conflict between teacher guidance and student dependence, stating that “*If I introduce GenAI to them, they will become overly dependent, lose their critical thinking ability, and become slaves to AI*”.

Most EFL teachers’ relatedness needs in the first round of interviews presented a combination of satisfaction and frustration. GenAI has enhanced teachers’ perceived competence in supporting student learning and facilitating teacher–student interaction, but concerns about students’ excessive reliance on GenAI also made teachers cautious about its educational implications. Therefore, teachers were beginning to explore new ways for GenAI to support teacher–student relationships, but their relationships remained shaped by tensions between educational responsibility and technological risks.

Most EFL teachers’ motivation for continued use of GenAI in the first round mainly demonstrated instrumental and early internalized characteristics, with efficiency, environmental adaptation, and teaching support as the main factors, while internal intrinsic motivation still remained at an emerging stage. Meanwhile, the needs for autonomy, competence, and relatedness showed a coexistence of satisfaction and frustration, supporting teachers’ ongoing exploration and experimentation with GenAI, but were not yet sufficient to facilitate deeper internalization of motivation.

### 4.2. Motivation Evolution and Basic Psychological Needs Experience in the Second Round of Interview (February–March 2026)

#### 4.2.1. Motivation Evolution in the Second Round of Interview (February–March 2026)

Although external regulation still exists, it no longer holds absolute dominance. Most university EFL teachers increasingly demonstrated identified regulation, integrated regulation, and intrinsic motivation. Furthermore, teaching empowerment, professional growth, core competencies, professional identity reshaping, and educational responsibility have emerged as the most prominent categories identified from the transcript (Table 4).

##### External Regulation

External regulation remained present but no longer the dominant motivational force. Almost all EFL teachers regarded GenAI as an indispensable support tool in improving work convenience. As T5 mentioned that “*GenAI has become a frequently used assistant in English email writing, as it can quickly generate expressions that are highly close to its own intentions and can be put into use with only minor adjustments*”. Moreover, some EFL teachers increasingly feel a sense of imperative and professional precarity to adapt to GenAI development as stated by T7’s statement of “*In the era of GenAI, if teachers lack understanding of GenAI, it will not only weaken their classroom leadership, but also lose the foundation for effective dialogue and guidance with students*”.

Teachers interviewed in the second round no longer only use GenAI as a tool to improve work efficiency, but gradually viewed it as a routine instructional support resource embedded in teaching practices, and paid increasing attention to keeping pace with advances in GenAI. Therefore, external regulation remained an important foundation for teachers’ continued use, but the motivation for continued use began to shift toward more autonomous and internalized forms of motivation.

##### Introjected Regulation

This subtheme consisted of two categories of concerns about professional obsolescence and student advancement and maintaining control through continuous adjustment. Most university EFL teachers expressed their concerns about professional obsolescence and student advancement. T9 confirmed this by stating: “*With the widespread application of AI now, I feel that this is an urgent matter… For myself, it is also a challenge, and I am worried that I will not keep up with the pace of the times… There will be a sense of urgency and a sense of crisis*”. What is more, some EFL teachers emphasized maintaining control through continuous adjustment in the interview: “*I will regard AI as a partner that needs guidance, guiding it to sort out logic through precise prompt words… Only through continuous interaction and debugging can I master the most suitable high-quality results for translation teaching*” (T5).

Most EFL teachers’ continued use of GenAI was characterized by a continued focus on professional competitiveness, classroom leadership, and students’ evolving learning needs, and teachers maintained a sense of professional control through continuous optimization of prompt design and refinement of human–GenAI collaboration strategies. Therefore, the internal pressure expressed by teachers had gradually shifted from technical adaptation to maintaining professional competence, and their degree of motivation internalization has further increased.

##### Identified Regulation

Identified regulation became more prominent in the second round of interviews. Most EFL teachers increasingly recognized the value of GenAI in providing personalized instructional support. T1 maintained, “*I mainly use it to design teaching activities, such as allowing students to practice speaking with GenAI and generate personalized reading materials*”. Some EFL teachers also use GenAI as a collaborator to optimize teaching design. As declared by T7, “*I think it is more like a collaborator at work. When designing the teaching of cross-border e-commerce English courses, with the assistance of Deepseek. I focused more on the theme and had a very innovative teaching experience*.”

Some EFL teachers in the second round perceived GenAI as an essential support resource for academic research, emphasizing its role in facilitating literature review, idea generation and research-related writing. This was confirmed by T3’s statement, “*GenAI can horizontally compare the differences in reliability indicators and parameters in different studies in a short period of time, which makes it realize that GenAI has become a ‘necessity’ in academic research*”.

Most EFL teachers interviewed in the second round showed clear recognition of the value of GenAI in personalized teaching, teaching innovation, and enhancing research capability. Some teachers also regarded GenAI as a necessary tool for academic research. Teachers’ recognition of the professional value of GenAI had significantly increased, and their motivation for continued use had further developed towards deeper motivational internalization and more autonomous forms of motivation.

##### Integrated Regulation

In integrated regulation, individuals not only recognize and identify with the value of an activity, but also view it as congruent with their core interests, values and self-identity (Ryan & Deci, 2020).

Some EFL teachers explicitly stated that they recognized the need to develop competence in using GenAI. As T12 argued that “*not being able to use GenAI is like not being able to use PPT or multimedia decades ago. The literacy empowered by GenAI technology is a key literacy for educators to keep up with the times and maintain core competitiveness.*” Meanwhile, some EFL teachers began to reflect on the perceptions of teachers’ professional roles. As mentioned in the interviews, “*GenAI cannot replace teachers, but it can provide teachers with a lot of references in interaction, better spreading knowledge to students. It is the most idea that teachers are very good at using GenAI and working perfectly with GenAI*” (T15).

Additionally, EFL teachers recognized that, in the context of widespread GenAI adoption, teachers are responsible for helping students use GenAI responsibly and critically. T7 maintained that “*If teachers know nothing about GenAI or deliberately avoid it, they will lose the opportunity to have a dialogue with students in the same discourse system, let alone guide students to use GenAI correctly for learning*”.

Integrated regulation was the most prominent newly emerging motivation type in the second round. Teachers not only consider competency in using GenAI as an essential capability for contemporary teachers, but also integrated GenAI use with evolving perceptions of teacher roles and educational responsibilities, assuming responsibility for guiding students to use GenAI responsibly and critically. Therefore, EFL teachers’ continued use of GenAI has extended beyond the recognition of its practical value in teaching, gradually integrating GenAI use into their professional values and teacher identity.

##### Intrinsic Motivation

Intrinsic motivation pertains to activities done “for their own sake,” or for their interest and enjoyment (Ryan & Deci, 2000a). Some EFL teachers began to demonstrate intrinsic interest in exploring GenAI. In this regard, T1 declared that “*In the Business English Negotiation course, I used GenAI to quickly generate language cases with different national styles, and student participation significantly increased. At that moment, I realized that GenAI can fully compensate for scenarios beyond my experience*”.

What is more, some EFL teachers experienced significant growth in competence in human–GenAI collaboration through continuous interaction, refinement and co-construction with GenAI. Several teachers mentioned that they began to pay more attention to optimizing prompt words, iterative interactions, comparing outputs generated by different GenAI, and filtering and correcting output content (T2, T3, T4, T7, T9, T10, T12, T13, T15). As T4 explained, “*when GenAI is unable to explain obscure theories, it will further consider how to obtain better answers through ‘more accurate prompt words*’”. It was further claimed that “*Faced with the challenges brought by AI, I will not give up, but rather start thinking about how to get better answers through more accurate prompts, which enhances my willingness to deeply master AI*” (T6).

Intrinsic motivation in the second round was further reflected in teachers’ continuous exploration and growth experiences in human–GenAI collaboration. Teachers not only enjoyed the process of applying GenAI to teaching innovation, but also gained a sense of achievement and satisfaction from continuously refined prompt words, engaging in iterative interactions, and collaboratively solving problems. Therefore, teachers’ continued interest in using GenAI had gradually shifted from the technology itself to the intrinsic experience of learning and competence development, reflecting a higher level of intrinsic motivation.

In the second round, although external regulation still exists, it had shifted from a primary driving force to a practical foundation for continued use. Meanwhile, identity regulation, integrated regulation, and intrinsic motivation had significantly increased, and teachers gradually recognized GenAI as an important component of modern teachers’ professional competence and integrated it into their educational responsibilities and professional identity.

#### 4.2.2. Basic Psychological Needs Experience in the Second Round of Interview (February–March 2026)

In the second round, the satisfaction and frustration of basic psychological needs no longer merely influenced whether EFL teachers continued using GenAI, but also influenced how they designed and adapted their teaching practices more proactively with GenAI. Particularly, need frustration did not weaken teachers’ willingness to continue using GenAI, but instead promoted deeper engagement through reflection and adjustment, as shown in Table 5.

##### Autonomy Experience

Autonomy need satisfaction has significantly increased as GenAI can not only help teachers complete routine teaching tasks, but also promote innovative teaching practices and enhance classroom management, enabling them to make more proactive decisions on “what to teach, how to teach, and how to adjust”. T7 argued that “*When designing my teaching, I asked Deepseek to provide me with many solutions and then gave targeted language instructions. With the help of AI, my teaching design became more focused on the theme, which left a deep impression on me as an innovative teaching experience*”.

Autonomy frustration became less prominent but did not completely disappear. Some university EFL teachers still experienced temporary constraints when faced with immature GenAI technology or teaching situations where GenAI provided limited support. However, this frustration is more understood as autonomy need frustration arising from the current technological limitations of GenAI. As T5 said, “*I really want to use it to make PPT, but the effect it produces is still unsatisfactory*”. T9 also expressed the need to continuously adjust the prompt words during use, saying “*when the result is not what I want, I will think if my instructions are not accurate enough, my description is not specific enough, or if my questioning style needs to be adjusted*”. Autonomous frustration was no longer mainly manifested as “not knowing how to use”, but more as “constantly adjusting to better use”.

Most EFL teachers’ experiences of autonomy need satisfaction in the second round have further evolved from “having opportunities to use GenAI” to “actively designing and adapting teaching practices”, revealing that teachers began to actively choose, adjust, and integrate GenAI based on their professional teaching judgments. At the same time, autonomy need frustration did not necessarily prevent teachers from continuing to use GenAI. Teachers may continue to explore possible solutions by adjusting their prompt strategies and usage approaches when encountering unsatisfactory GenAI-generated outputs.

##### Competence Experience

Competence need satisfaction has become one of the core forces promoting deeper motivational internalization. University EFL teachers are increasingly aware that GenAI can help them broaden their knowledge and cognitive perspectives, enhance academic research competence, optimize instructional design, and strengthen human–GenAI collaboration. This enhanced sense of competence satisfaction was reflected in teachers’ perceptions of being “more professional, more in control, and better able to handle complex tasks”. T3 mentioned the ability of GenAI to handle complex tasks by arguing that “*faced with dozens of test papers involving complex statistics, GenAI was able to help me horizontally compare the reliability indicators and parameter differences of different studies within seconds*”. T10 emphasized the improvement of human–machine collaboration ability, saying “*This experience has given me a clearer understanding of how to use it, and using it correctly can help me efficiently integrate resources and expand my thinking*”.

Some university EFL teachers still experienced frustration when faced with GenAI’s inability to explain complex theories, address complex rhetorical issues, as well as its tendency to generate inaccurate outputs. However, this frustration no longer primarily served as a hindrance, but was instead transformed into a reaffirmation of teachers’ professional value and a pursuit of more advanced human–GenAI collaboration competence. T3 declared that “*I once felt frustrated when AI was unable to explain an obscure language testing theory*”. Optimizing the output results through debugging was confirmed by a teacher, who asserted that “*In the process of using AI, when it cannot meet the requirements or makes mistakes, I usually do not give up, but constantly interact and adjust to produce high-quality results*” (T15).

Most EFL teachers’ competence needs satisfaction has further evolved from “improving task completion efficiency” to “completing complex tasks with greater professionalism and confidence”, becoming an important force in promoting deeper motivation to continue the use of GenAI. At the same time, competence frustration is no longer mainly manifested as usage barriers, but rather prompts teachers to improve their own abilities through refining prompt words, engaging in repeated interactions with GenAI, and manually evaluating outputs when facing inaccurate GenAI-generated outputs or difficulties in handling complex tasks.

##### Relatedness Experience

Relatedness need satisfaction has significantly deepened. Most teachers no longer experience relatedness support solely through “more detailed feedback”; instead, they increasingly understand relatedness need satisfaction from the perspective of sharing the same GenAI-mediated educational environment with students and taking greater responsibility for guiding students’ appropriate use of GenAI. As T7 stated, “*If teachers know nothing about AI or deliberately avoid it, we will lose the opportunity to converse with students in the same discourse system*”. T13 has also reconfirmed this point, stating that “*without mastering GenAI, we cannot identify AI generated content in assessments, let alone guide students to produce authentic language in the era of artificial intelligence*”.

Relatedness need frustration was manifested as temporary discomfort and uncertainty experienced by university EFL teachers when adapting to students’ increasing use of GenAI. As noted by T5, “*At the beginning, teachers may feel some discomfort when students use GenAI, but now they have fully adapted and will adjust some teaching strategies accordingly*”. Another teacher expressed their anxiety about the teacher role by stating: “*I think there is a problem with the teaching subject here…This subject has become blurred and blurred*”.

Relatedness need in the second round showed a trend of maintaining teacher–student relationships toward assuming greater educational responsibility. Most teachers placed more emphasis on maintaining a shared understanding with students in the era of GenAI, and addressed challenges brought by students’ widespread use of GenAI through adjustments in teaching strategies. Therefore, relatedness need satisfaction has further developed from facilitating interaction toward maintaining meaningful teacher–student connections and fulfilling educational guidance responsibilities. Meanwhile, relatedness need frustration did not directly prevent teachers from using GenAI. Instead, some teachers began to reconsider their guidance roles and educational responsibilities when faced with issues such as students’ excessive reliance on GenAI.

In the second round, EFL teachers’ satisfaction of basic psychological needs became more stable, and needs frustration was increasingly perceived not as a constraint on GenAI use, but in some cases, as a catalyst for strategy adjustment and the development of human–GenAI collaboration competence.

### 4.3. Longitudinal Comparison and Motivation Evolution of Two Rounds of Interviews

It should be mentioned that the longitudinal evolution presented in this study mainly reflected the thematic pattern changes at the level of the participant group, rather than suggesting that all individuals experienced the same motivational transformation. The comparison of two rounds of thematic analysis focused on the emergence, expansion, and changes in the manifestation and interpretation of motivational themes across different time points, and presented this evolution through the common patterns and variations among participants. Therefore, the “motivation evolution” examined in this study should be understood as a longitudinal thematic trend at the sample level rather than a linear developmental trajectory experienced by each participant.

Based on two rounds of interviews, it can be found that the motivation for EFL teachers’ motivation for continued use of GenAI was not simply a shift from one motivational type to another, but rather a gradual evolution from external regulation and tool-oriented use toward autonomous internalization and professional integration (Table 6). In the first round, teachers mainly focused on environmental adaptation, work efficiency, and teaching support, whereas in the second round, they placed greater emphasis on the value of GenAI for teaching innovation, professional growth, and professional identity. Although external regulation still existed, it shifted from a primary driving force to a practical foundation for continued use. At the same time, identity regulation, integrated regulation, and intrinsic motivation significantly increased, and teachers gradually recognized GenAI as an important component of contemporary teachers’ professional competencies, integrating it into their professional identity and educational responsibilities. Overall, teachers’ motivation for continued use of GenAI demonstrated a gradual internalization pattern from “environmental adaptation and tool support” toward “teaching empowerment, professional growth, and professional identity”.

As can be seen in Table 7, the results showed that the satisfaction and frustration of basic psychological needs may represent psychological factors underlying the evolution of university EFL teachers’ motivation to continue using GenAI. Autonomous need satisfaction was generally associated with teachers’ transition from using GenAI to completing routine tasks toward actively organizing and regulating teaching practices. Competence need satisfaction was associated with teachers’ shift from focusing on improving efficiency to enhancing professional and competence and human–GenAI collaboration. Relatedness need satisfaction encouraged EFL teachers to pay greater attention to student guidance and instructional support rather than focusing solely on feedback optimization.

It is worth noting that the role of basic psychological needs frustration changed across the two rounds of interviews. In the first round, EFL teachers’ insufficient technical proficiency, unsatisfactory GenAI output, and worries about students’ excessive dpendence on GenAI constrained their further exploration of GenAI. However, by the second round, teachers came to frame these frustrating experiences as opportunities to improve human–GenAI collaboration via reflection and adaptive adjustment. For example, when encountering GenAI’s inability to handle complex tasks, teachers reported that they would refine prompts, draw upon their professional knowledge, and manually evaluate or correct generated outputs rather than discontinuing their use of GenAI. Therefore, the role of need frustration gradually shifted from an early barrier to continued use toward a developmental opportunity that promoted the improvement of human–GenAI collaboration capabilities through reflection and adaptation.

Overall, EFL teachers’ continued use of GenAI demonstrated a gradual evolution from “environmental adaptation and tool support” to “teaching empowerment, professional growth and professional identity integration”. The central process was not the replacement of one motivation by another, but rather the gradual internalization and professional integration of motivation through sustained engagement with GenAI.

## 5. Discussion

### 5.1. Motivation Evolution for University EFL Teachers’ Continued Use of GenAI

The findings from the two rounds of interviews revealed that university EFL teachers’ motivation for continued use evolved from instrumental and externally regulated use toward value identification, professional integration, and increasingly autonomous engagement. Rather than following a simple linear progression along the SDT motivational continuum, different types of motivation coexisted and were continually reorganized through teachers’ ongoing practice. It is consistent with Howard et al. (2017), who demonstrated that motivational regulations form a continuum rather than discrete categories. This finding supports SDT’s view that motivation is gradual and context-dependent (Ryan & Deci, 2000b, 2020) and extends Howard et al.’s (2017) continuum framework by providing longitudinal evidence that motivational regulations remain dynamic and mutually interactive during continued technology use.

Firstly, instrumental value constitutes the starting point rather than the endpoint of motivational development. During the initial stage, teachers primarily continued using GenAI because it improved work efficiency, reduced repetitive tasks, and facilitated lesson preparation and academic writing. These findings are consistent with the Technology Acceptance Model (TAM), which emphasizes the role of perceived usefulness in technology acceptance (Davis, 1989; Venkatesh et al., 2003; Salas, 2016), and also echoed recent studies showing that teachers evaluate GenAI based on practical benefits such as convenience and efficiency improvement (Soliman et al., 2024; Bamasoud et al., 2025). However, our study further suggested that perceived usefulness, which helps explain teachers’ initial adoption to continue using GenAI, was insufficient to account for how and why their motivation becomes deeply internalized over time. Teachers’ early engagement remained largely task-oriented and was constrained by technological competence and contextual limitations, indicating that instrumental value serves primarily as the foundation for subsequent motivational internalization.

Secondly, motivational development is characterized by the coexistence and dynamic reorganization of controlled and autonomous motivation. As teachers accumulated experience, identified regulation became increasingly salient, while external and introjected regulations did not disappear. Instead, teachers simultaneously recognized GenAI’s instructional and research value and experienced continuing professional pressure arising from rapid technological change. This finding aligns with SDT, which suggests that different types of motivation may coexist and dynamically interact rather than simply replace one another (Ryan & Deci, 2020). It also echoes previous research highlighting the non-linear development of motivation (Luqman et al., 2018; Vansteelandt et al., 2020; Chiu et al., 2024). However, the findings extend previous research by demonstrating this pattern within the context of continued use of GenAI. Importantly, the present findings do not suggest that professional anxiety itself promotes motivational internalization. Rather, they indicate that teachers may maintain continued engagement while negotiating both professional values and external demands, highlighting the contextual complexity of motivational development.

Furthermore, continued use gradually becomes embedded within teachers’ professional growth and identity construction. Beyond recognizing GenAI as a useful instructional tool, teachers increasingly regarded GenAI competence as an integral component of effective teaching, professional development, and educational responsibility. This finding aligns with teacher identity research, which conceptualizes professional identity as continuously reconstructed through professional experience and changing educational contexts (Beijaard et al., 2004; Akkerman & Meijer, 2010). It also supports Avalos’ (2010) view that professional development involves not only acquiring new instructional competencies but also redefining teachers’ professional roles. Our findings extend these perspectives by showing that continued interaction with GenAI encourages teachers to move from technology users toward human–GenAI collaborators while assuming greater responsibility for guiding students’ appropriate use of GenAI.

Additionally, intrinsic motivation gradually emerges through professional learning and human–GenAI collaboration rather than from initial curiosity alone. Although some teachers initially explored GenAI out of curiosity, intrinsic motivation became increasingly associated with the enjoyment of solving complex pedagogical problems, refining prompts, and improving collaborative competence through sustained interaction with GenAI. Consistent with studies highlighting the role of human–AI collaboration in promoting teacher learning (Meniado, 2024; S. Chen & Wang, 2024), this finding suggests that intrinsic motivation is better understood as an outcome of continued professional growth rather than the initial driver of technology use.

Overall, this finding extends the application of SDT in the context of technology integration by demonstrating that university EFL teachers’ motivation for continued use of GenAI does not simply develop along a linear continuum from external regulation to intrinsic motivation, but it involves the coexistence, expansion, and reorganization of multiple motivational orientations through continued practice. Initial instrumental value provides the foundation for continued engagement, value recognition encourages broader teaching and research applications, and professional experience, role reflection, and human–GenAI collaboration further facilitate the integration of GenAI into teachers’ professional value systems. Therefore, this study advances existing research beyond whether teachers accept GenAI toward understanding how teachers reinterpret its value and develop more autonomous orientations during continued use.

### 5.2. Influence of Satisfaction and Frustration of Basic Psychological Needs on Motivation Evolution

This finding revealed that the dynamic experience of basic psychological needs constitutes an important psychological foundation underlying teachers’ motivation for continued use of GenAI, which echoes the SDT perspective that psychological needs of autonomy, competence and relatedness provide an important foundation for motivation internalization (Deci & Ryan, 2000; Ryan, 2023). Meanwhile, this study further indicated that in the context of GenAI, psychological needs do not function in a static manner, but demonstrate distinct dynamic characteristics as teachers accumulate experience with GenAI. The longitudinal findings showed that teachers’ needs experience has gradually developed from partial need satisfaction accompanied by substantial frustration in the first round to a state of coexistence of high-level needs satisfaction and moderate needs frustration in the second round. This transformation corresponded to the gradual shift in teachers’ motivation from instrumental use to professional integration.

First, this study indicated that psychological needs satisfaction and needs frustration may exert dual-pathway influences on motivational evolution during continued use of GenAI, which corroborates SDT’s dual-process model (Ryan, 2023). What is more, this study further revealed that need satisfaction and need frustration are not simply opposing relationships, but jointly constitute the psychological experience of teachers during continued use of GenAI, which was supported by B. Chen et al. (2014), who argued that need satisfaction and need frustration represent separate psychological dimensions rather than opposite poles. Furthermore, need satisfaction continued to facilitate motivational internalization across different stages, which aligns with the classic SDT proposition that need satisfaction could facilitate the development of autonomous motivation (Ryan, 2023; Alamer & Ai Khateeb, 2023). Specifically, autonomy need gradually evolved from task processing convenience to teaching control sense, and competence need shifted from efficiency improvement to human–GenAI collaboration capabilities, and relatedness need evolved from providing interactive support to fostering educational responsibility and professional identity recognition. These findings extend previous studies by showing that different psychological needs do not necessarily develop simultaneously, but contribute to motivational internalization at different levels, including teaching practice, professional development, and professional identity as teachers’ practical experience continues to accumulate, which aligns with Vansteenkiste and Ryan (2013), who found that need satisfaction provided psychological support for motivational internalization.

Third, this longitudinal study further explained that need frustration does not invariably have a negative influence on the development of autonomous motivation, but offers an opportunity to enhance their human–GenAI cooperation through iterative reflection and adjustment. At the initial stage, need frustration, including limited technological competence, institutional constraints, and concerns about students’ over-dependence on GenAI, did indeed hinder motivation deepening. This finding aligns with the conclusion of existing research suggesting that need frustration may negatively influence the development of more autonomous motivation (Warburton et al., 2019). However, this study further suggests in the second round that when teachers had developed a high level of autonomy, competence and relatedness satisfaction, they still experienced a certain degree of need frustration due to unstable GenAI output, limited processing of complex tasks, and changing perceptions of teachers’ roles, which is consistent with previous research suggesting that need satisfaction and need frustration can coexist (Ryan, 2023; Rodrigues et al., 2021; Krijgsman et al., 2025). However, need frustration was increasingly manifested as a continuous reflection on technological boundaries and professional development rather than as a direct reduction in willingness to continue using, as observed in the first round. For some teachers, encountering inaccurate AI output, limited technical capabilities, or excessive student dependence did not directly reduce their willingness to use it. Instead, these challenges encouraged them to reconsider problems, adjust prompt strategies, optimize teaching practices, and reflect on their professional roles. Therefore, within this sample, need frustration may be reinterpreted as an opportunity for reflection, adjustment, and learning under specific conditions, accompanied by deeper engagement with GenAI. However, such transformation is not universal and may depend on teachers’ coping strategies and specific usage contexts.

Finally, this study extended the application of SDT in educational technology contexts by revealing that EFL teachers’ experiences may involve a dynamic process in which need frustration is interpreted as an opportunity for reflection and adjustment, potentially contributing to enhanced need satisfaction over time. This finding indicates that teachers’ motivation for continued use of GenAI is not necessarily driven by the barrier-free technology, but rather by the transformation from tool users to human–GenAI collaborative designers through continuous reflection and adjustments. Therefore, within GenAI-supported teaching contexts, psychological need experience may be better understood as dynamic and iterative processes rather than simple linear relationships underlying motivational development. This finding contributes to extending SDT in educational technology research and provides a new perspective for understanding how EFL teachers’ continued use of GenAI may be associated with professional growth and professional identity formation.

This study makes three theoretical contributions. First, by adopting a longitudinal qualitative design, it extends the application of Self-Determination Theory (SDT) to GenAI-supported EFL education, demonstrating that teachers’ motivation and basic psychological needs evolve dynamically rather than remaining static. The findings show how autonomy, competence, and relatedness are continuously reconstructed as teachers progress from externally regulated use toward more autonomous engagement. Secondly, this study suggests that EFL teachers’ experiences of need frustration are not invariably demotivating. Instead, some teachers continued to deepen their GenAI use through reflection and adjustment, suggesting that its motivational implications depend on individual interpretations and coping strategies rather than functioning as a uniform barrier. Thirdly, this study proposes a motivational evolution pathway from instrumental technology use to professional identity integration. It demonstrates that teachers’ continued use of GenAI develops from efficiency-oriented motivation toward teaching empowerment, professional growth, and identity construction, thereby linking technology integration with teacher professional development and offering a conceptual framework for future research on GenAI and teacher development.

The findings also provide practical implications for EFL teachers, policymakers and GenAI developers. For EFL teachers, teacher training programs may benefit from emphasizing the satisfaction of psychological needs to foster continued motivation for GenAI use. Such training should not only focus on reducing their workload, but also improving human–GenAI collaboration skills and their ability to evaluate GenAI-generated outcomes critically. For education policymakers, the findings suggest the importance of creating supportive environments fostering teachers’ psychological well-being during technology integration, including providing professional development opportunities, establishing clear guidelines for GenAI use, encouraging collaborative learning communities and recognizing innovative teaching practices involving GenAI. For GenAI developers, the findings highlight the importance of designing teacher-centered GenAI suitable for EFL teaching scenarios, further promoting the widespread application of GenAI in EFL education. Additionally, the findings also suggest that frustration encountered during the continued use of GenAI may represent opportunities for reflection rather than merely barriers to technology use. EFL teachers may benefit from reflection and adjustment during continued use of GenAI to better understand the sources of these challenges and potentially transform them into opportunities for professional learning and growth.

## 6. Limitations and Suggestions for Future Research

### 6.1. Limitations

Although this study has provided valuable insights, several limitations should be acknowledged. Firstly, this study adopted a longitudinal qualitative design, which enabled an in-depth exploration of the evolution of teachers’ motivation and their experiences of psychological need satisfaction and frustration. However, the qualitative nature of the study limits the ability to statistically examine the relationships among basic psychological needs satisfaction, need frustration, and motivational internalization. Therefore, the findings should be interpreted primarily as theoretically informative explanations of teachers’ motivational development, and their generalizability requires further validation through quantitative approaches. Second, the sample size of this study was relatively small, involving only 15 university EFL teachers in China. Although the longitudinal interviews generated rich process-oriented data, the findings remain embedded within specific educational and cultural contexts. Therefore, the applicability of the findings to EFL teachers in different countries, educational levels, and disciplinary contexts should be considered with caution. Finally, this study primarily examined the motivational evolution and psychological need experiences underlying continued use of GenAI from teachers’ perspectives. Although participants frequently discussed teacher–student interactions related to GenAI use, the study did not incorporate students’ perspectives. Consequently, it remains unclear how teachers and students may jointly shape motivational changes and technology-related practices during the continued use of GenAI in educational contexts.

### 6.2. Suggestions for Future Research

Future research may proceed in several directions. First, based on the qualitative insights generated from this study, future studies could develop and validate relevant measurement instruments and employ quantitative approaches, such as questionnaire surveys and structural equation modeling (SEM), to further examine the relationships among need satisfaction, need frustration, and motivational internalization. Second, future research could extend the participant pool across diverse educational contexts and cultural settings to further examine the transferability and generalizability of the findings. A larger and more diverse sample may also provide deeper insights into how EFL teachers’ motivations for continued GenAI use vary across different groups and contexts. Finally, future studies could incorporate both teacher and student perspectives to explore how GenAI jointly shapes learning experiences, teacher–student interactions, and motivational processes. Such an integrated perspective may contribute to developing a more comprehensive theoretical framework for understanding the sustained use of GenAI in educational contexts.

## 7. Conclusions

This longitudinal qualitative study explored the evolution of university EFL teachers’ motivation and psychological need experiences underlying continued GenAI use from the perspective of Self-Determination Theory (SDT). The findings indicate that teachers’ motivation is not static but evolves from environmental adaptation, work efficiency, and teaching support toward teaching empowerment, professional growth, and professional identity. Rather than following a simple linear progression, this evolution involves the coexistence, reorganization, and gradual internalization of multiple motivational orientations through continued engagement with GenAI.

The study further demonstrates that dynamic changes in basic psychological needs provide an important psychological foundation for this motivational evolution. As teachers accumulated experience, satisfaction of autonomy, competence, and relatedness generally increased, while the meaning of need frustration became increasingly context-dependent. For some teachers, frustration did not simply undermine continued use but was interpreted as an opportunity for reflection, strategy adjustment, professional learning, and deeper engagement with GenAI. These findings suggest that the relationship between need satisfaction, need frustration, and motivational development is dynamic rather than static.

In conclusion, these findings offer a contextualized extension of SDT in the education context of GenAI by providing preliminary, contextually grounded insights into how motivational internalization and psychological need experiences may unfold during teachers’ continued use of GenAI. It suggests that teachers’ continued use of GenAI should be understood not merely as continued technology adoption, but as a dynamic process of motivational internalization, evolving psychological need experiences, professional growth, and identity development. These findings contribute to a deeper understanding of the psychological need experience underlying teachers’ continued engagement with GenAI and provide implications for supporting teacher professional development and the sustainable integration of GenAI in language education.

## Figures and Tables

**Table 1:** Participant demographic details (*N* = 15).

Background Information	Category	Count	Percentage
Age	25–30	5	33.3%
	31–40	5	33.3%
	41–50	5	33.3%
Gender	Male	6	40%
	Female	9	60%
Academic degree	PhD	5	33.3%
	Master’s	10	66.7%
Institution	University	8	53%
	College	7	47%
Disciplinary	English Translation	5	33.3%
	Business English	5	33.3%
	General English	5	33.3%

**Table 2:** Motivation coding of university EFL teachers’ continued use of GenAI in the first round of interviews.

Theme	Subtheme	Category
Motivation types	External Regulation (strong)	Improve work efficiency
		Deal with teaching and writing tasks
	Introjected Regulation (moderate)	Keep up with the times
		Follow peer choices
	Identified Regulation (moderate)	Spark new ideas and broaden perspectives
		Enhance teaching design and scientific research
	Intrinsic Regulation (weak)	Possess curiosity about new technology
		Hold interest in exploring the different usages

**Table 3:** Psychological need coding of EFL teachers’ continued use of GenAI in the first round of interviews.

Theme	Subtheme	Category
Basic Psychological Needs	Autonomy satisfaction	Prepare teaching materials flexibly Acquire information efficiently Design classroom assignments
	Autonomy frustration	Lack proficiency in advanced applications Face limitations in mobile device usage
	Competence satisfaction	Improve working efficiency Develop teaching plans Serve as an initial academic assistant
	Competence frustration	Struggle with GenAI tools Receive inaccurate generated outputs Fail to execute advanced work
	Relatedness satisfaction	Provide detailed feedback Promote classroom discussions Enhance student participation
	Relatedness frustration	Induce students’ over-dependence on GenAI Obtain untrue outcomes Diminish the authenticity of learning experiences

**Table 4:** Motivational coding of EFL teachers’ continued use of GenAI in the second round of interview.

Theme	Subtheme	Category
Motivational types	External Regulation (medium)	Facilitate work convenience Adapt to GenAI development
	Introjected Regulation (moderate)	Concern professional obsolescence and student advancement
		Maintain control through continuous adjustment
	Identified Regulation (strong)	Provide personalized teaching support
		Enhance scientific research capabilities
	Integrated Regulation (very strong)	Recognize the need to develop competence in using GenAI
		Reshaping perceptions of teachers’ professional roles
		Guiding students’ use of GenAI
	Intrinsic Motivation (moderate–strong)	Show intrinsic interest to explore GenAI
		Strategies for working with GenAI

**Table 5:** Psychological need coding of EFL teachers’continued use of GenAI in the second round of interviews.

Theme	Subtheme	Category
Basic Psychological Needs	Autonomy satisfaction	Innovate teaching methods Adjust teaching content dynamically Enhance classroom management
	Autonomy frustration	Encounter unstable GenAI output performance Adjust prompt strategies continuously
	Competence satisfaction	Boost personal teaching innovation; Improve academic research quality Enhance human-AI collaboration experiences
	Competence frustration	Fail to handle complex work tasks Receive incorrect or unstable outputs Require manual correction and verification
	Relatedness satisfaction	Share the same context with students Deliver better responses to students’ needs Maintain the role of teaching guidance
	Relatedness frustration	Feel discomfort for students using AI Worry about the authenticity of teaching subjects

**Table 6:** Comparative analysis of motivation evolution across two rounds of interviews.

Motivation Types	First Round	Second Round	Evolution
External regulation	Strong	Medium	↓
Introjected regulation	Moderate	Moderate	↑
Identified regulation	Moderate	Strong	↑
Integrated regulation	Minimal	Very strong	↑↑
Intrinsic motivation	Weak	Moderate–strong	↑

Note: ↓ = decrease in motivational strength from the first-round interview to the second-round interview; ↑ = increase in motivational strength; ↑↑ = substantial increase in motivational strength.

**Table 7:** A comparison of psychological need experience and motivation evolution across two rounds of interviews.

Theme	Subtheme	First Round	Second Round	Motivation Evolution
Basic Psychological Needs	Autonomy satisfaction	weak	strong	External regulation → Identified regulation
	Autonomy frustration	strong	medium	External regulation → introjected/identified regulation
	Competence satisfaction	strong	stronger	Identified regulation → Integrated regulation → Intrinsic regulation
	Competence frustration	strong	Transformed into iterative reflection, and adjustment	Introjected regulation → Integrated regulation
	Relatedness satisfaction	weak	strong	Identified regulation → integrated regulation
	Relatedness frustration	weak	strong	Introjected regulation → Integrated regulation

## Data Availability

The datasets generated and analyzed during the current study are available from the corresponding author on reasonable request. The data are not publicly available due to privacy and ethical restrictions.

## Abbreviations

The following abbreviations are used in this manuscript:

SDT	Self-Determination Theory
GenAI	Generative Artificial Intelligence
TAM	Technology Acceptance Model
UTAUT	Unified Theory of Technology Acceptance and Use

## Appendix A

**Interview Questions**
  **Background Information:**
  1. Age:
  2. Gender:
  3. Educational Background:
  4. Research field:
  5. Institution
  **Open-Ended Questions in the first round of interview:**
  *Would you like to describe your primary motivations for using GenAI? Please elaborate on at least three key motivations with detailed explanations.*
  *Do you feel that Continued use of GenAI is helping you to become more autonomous in your work? Can you give one example at least, please.*
  *Do you feel that continued use of GenAI is helping you to become a more competent and more effective in your work? Can you give one example at least, please.*
  *Do you feel that continued use of GenAI is changing the way you interact with your students? Can you give one example at least, please.*
  *Have your motivations for using GenAI evolved over time? If so, what factors contributed to this change?*
  **Open-Ended Questions in the second round of interview:**
  *When your basic psychological needs are frustrated or challenged by the limitations of GenAI (for example, when GenAI fails to accurately interpret or resolve complex grammatical questions), would you tend to give up using GenAI or actively seek alternative solutions?*
  *How did this psychological fluctuation influence your willingness and persistence for your continued use of GenAI?*
  *To what extent do you consider using GenAI as an essential professional quality for modern EFL teachers?*
